# Supplementary material for: Role and mechanism of NCAPD3 in promoting malignant behaviors in gastric cancer
Source: Front Pharmacol. 2024 Apr 22;15:1341039. doi: 10.3389/fphar.2024.1341039 (PMC11070777; doi:10.3389/fphar.2024.1341039)
Supplement: Supplementary file 11 [file DataSheet2.ZIP › GSEA/GO_CC/my_analysis.Gsea.1599461408869/GO_TRANSFERASE_COMPLEX.html]

Details for gene set GO\_TRANSFERASE\_COMPLEX[GSEA]

|  || Dataset | filtered\_dataset.sample\_info.cls#WT\_versus\_NCAPD3\_MUT |
| Phenotype | sample\_info.cls#WT\_versus\_NCAPD3\_MUT |
| Upregulated in class | WT |
| GeneSet | GO\_TRANSFERASE\_COMPLEX |
| Enrichment Score (ES) | 0.19311297 |
| Normalized Enrichment Score (NES) | 1.2465037 |
| Nominal p-value | 0.1749226 |
| FDR q-value | 0.720783 |
| FWER p-Value | 1.0 |
Table: GSEA Results Summary

  

Fig 1: Enrichment plot: GO\_TRANSFERASE\_COMPLEX      
 Profile of the Running ES Score & Positions of GeneSet Members on the Rank Ordered List

  

| SYMBOL | TITLE | RANK IN GENE LIST | RANK METRIC SCORE | RUNNING ES | CORE ENRICHMENT || 1 | 2072 | ERCC4 | 7 | 1.130 | 0.0202 | Yes |
| 2 | 23248 | RPRD2 | 33 | 0.977 | 0.0235 | Yes |
| 3 | 57542 | KLHL42 | 59 | 0.876 | 0.0245 | Yes |
| 4 | 23338 | JADE2 | 71 | 0.855 | 0.0355 | Yes |
| 5 | 23054 | NCOA6 | 77 | 0.838 | 0.0506 | Yes |
| 6 | 3667 | IRS1 | 83 | 0.821 | 0.0654 | Yes |
| 7 | 55006 | TRMT61B | 101 | 0.782 | 0.0703 | Yes |
| 8 | 26272 | FBXO4 | 133 | 0.767 | 0.0643 | Yes |
| 9 | 51434 | ANAPC7 | 166 | 0.710 | 0.0563 | Yes |
| 10 | 90025 | UBE3D | 167 | 0.710 | 0.0723 | Yes |
| 11 | 23590 | PDSS1 | 175 | 0.701 | 0.0828 | Yes |
| 12 | 57325 | CSRP2BP | 176 | 0.698 | 0.0986 | Yes |
| 13 | 55070 | DET1 | 234 | 0.648 | 0.0704 | Yes |
| 14 | 23001 | WDFY3 | 240 | 0.642 | 0.0811 | Yes |
| 15 | 1161 | ERCC8 | 255 | 0.625 | 0.0847 | Yes |
| 16 | 152485 | ZNF827 | 268 | 0.618 | 0.0896 | Yes |
| 17 | 25852 | ARMC8 | 307 | 0.588 | 0.0743 | Yes |
| 18 | 26259 | FBXW8 | 318 | 0.583 | 0.0799 | Yes |
| 19 | 1021 | CDK6 | 324 | 0.579 | 0.0892 | Yes |
| 20 | 55697 | VAC14 | 329 | 0.576 | 0.0993 | Yes |
| 21 | 4297 | KMT2A | 337 | 0.573 | 0.1069 | Yes |
| 22 | 5356 | PLRG1 | 338 | 0.573 | 0.1199 | Yes |
| 23 | 2342 | FNTB | 344 | 0.570 | 0.1290 | Yes |
| 24 | 79184 | BRCC3 | 348 | 0.569 | 0.1395 | Yes |
| 25 | 2965 | GTF2H1 | 356 | 0.563 | 0.1470 | Yes |
| 26 | 1387 | CREBBP | 387 | 0.544 | 0.1367 | Yes |
| 27 | 54554 | WDR5B | 395 | 0.539 | 0.1436 | Yes |
| 28 | 54994 | GID8 | 415 | 0.526 | 0.1412 | Yes |
| 29 | 8607 | RUVBL1 | 451 | 0.506 | 0.1263 | Yes |
| 30 | 200933 | FBXO45 | 456 | 0.500 | 0.1346 | Yes |
| 31 | 27097 | TAF5L | 461 | 0.498 | 0.1428 | Yes |
| 32 | 10785 | WDR4 | 465 | 0.495 | 0.1517 | Yes |
| 33 | 5295 | PIK3R1 | 467 | 0.493 | 0.1621 | Yes |
| 34 | 79960 | JADE1 | 468 | 0.493 | 0.1732 | Yes |
| 35 | 23468 | CBX5 | 484 | 0.485 | 0.1729 | Yes |
| 36 | 140775 | SMCR8 | 487 | 0.480 | 0.1822 | Yes |
| 37 | 8454 | CUL1 | 492 | 0.477 | 0.1900 | Yes |
| 38 | 11060 | WWP2 | 503 | 0.471 | 0.1931 | Yes |
| 39 | 7334 | UBE2N | 546 | 0.449 | 0.1717 | No |
| 40 | 6632 | SNRPD1 | 563 | 0.440 | 0.1696 | No |
| 41 | 56339 | METTL3 | 613 | 0.417 | 0.1421 | No |
| 42 | 10445 | MCRS1 | 617 | 0.414 | 0.1492 | No |
| 43 | 22847 | ZNF507 | 618 | 0.413 | 0.1585 | No |
| 44 | 51728 | POLR3K | 646 | 0.401 | 0.1473 | No |
| 45 | 29890 | RBM15B | 651 | 0.400 | 0.1533 | No |
| 46 | 79269 | DCAF10 | 668 | 0.394 | 0.1502 | No |
| 47 | 55958 | KLHL9 | 710 | 0.370 | 0.1277 | No |
| 48 | 84172 | POLR1B | 718 | 0.365 | 0.1307 | No |
| 49 | 122809 | SOCS4 | 726 | 0.360 | 0.1335 | No |
| 50 | 899 | CCNF | 727 | 0.360 | 0.1416 | No |
| 51 | 84148 | KAT8 | 757 | 0.344 | 0.1276 | No |
| 52 | 80218 | NAA50 | 782 | 0.332 | 0.1171 | No |
| 53 | 11091 | WDR5 | 822 | 0.304 | 0.0946 | No |
| 54 | 26121 | PRPF31 | 825 | 0.301 | 0.0999 | No |
| 55 | 8835 | SOCS2 | 840 | 0.275 | 0.0956 | No |
| 56 | 10856 | RUVBL2 | 847 | 0.257 | 0.0968 | No |
| 57 | 4008 | LMO7 | 909 | -0.335 | 0.0585 | No |
| 58 | 8881 | CDC16 | 963 | -0.383 | 0.0273 | No |
| 59 | 901 | CCNG2 | 998 | -0.405 | 0.0109 | No |
| 60 | 2146 | EZH2 | 1001 | -0.406 | 0.0186 | No |
| 61 | 51343 | FZR1 | 1005 | -0.407 | 0.0255 | No |
| 62 | 51646 | YPEL5 | 1014 | -0.413 | 0.0288 | No |
| 63 | 10616 | RBCK1 | 1042 | -0.438 | 0.0184 | No |
| 64 | 26260 | FBXO25 | 1051 | -0.447 | 0.0225 | No |
| 65 | 5111 | PCNA | 1077 | -0.460 | 0.0141 | No |
| 66 | 196394 | AMN1 | 1079 | -0.461 | 0.0237 | No |
| 67 | 5565 | PRKAB2 | 1094 | -0.474 | 0.0239 | No |
| 68 | 5277 | PIGA | 1126 | -0.500 | 0.0118 | No |
| 69 | 64795 | RMND5A | 1142 | -0.508 | 0.0120 | No |
| 70 | 1032 | CDKN2D | 1156 | -0.519 | 0.0140 | No |
| 71 | 146330 | FBXL16 | 1180 | -0.549 | 0.0091 | No |
| 72 | 8535 | CBX4 | 1184 | -0.555 | 0.0193 | No |
| 73 | 8408 | ULK1 | 1243 | -0.622 | -0.0102 | No |
| 74 | 114907 | FBXO32 | 1248 | -0.625 | 0.0009 | No |
| 75 | 8651 | SOCS1 | 1272 | -0.661 | -0.0015 | No |
| 76 | 7994 | KAT6A | 1279 | -0.666 | 0.0090 | No |
| 77 | 54800 | KLHL24 | 1287 | -0.677 | 0.0191 | No |
| 78 | 85417 | CCNB3 | 1299 | -0.700 | 0.0266 | No |
| 79 | 493861 | EID3 | 1315 | -0.729 | 0.0318 | No |
| 80 | 255488 | RNF144B | 1362 | -0.834 | 0.0160 | No |
| 81 | 4734 | NEDD4 | 1369 | -0.856 | 0.0308 | No |
Table: GSEA details [plain text format]

  

Fig 2: GO\_TRANSFERASE\_COMPLEX      
 Blue-Pink O' Gram in the Space of the Analyzed GeneSet

  

Fig 3: GO\_TRANSFERASE\_COMPLEX: Random ES distribution      
 Gene set null distribution of ES for **GO\_TRANSFERASE\_COMPLEX**
